# Supplementary material for: Optimization of carbon and energy utilization through differential translational efficiency
Source: Nat Commun. 2018 Oct 26;9:4474. doi: 10.1038/s41467-018-06993-6 (PMC6203783; doi:10.1038/s41467-018-06993-6)
Supplement: Supplementary file 1 — Supplementary Information [file 41467_2018_6993_MOESM1_ESM.pdf]

SUPPLEMENTARY INFORMATION

Al-Bassam et al.

RNA-seq data

Library	Type	Total reads sequenced[1]	Total reads aligned	Efficiency %[2]	Coverage	#Paired-end reads CDS
CO1	Paired-end	168,650,056	162,574,412	96.40	3511x	77,871,818
CO2	Paired-end	180,003,754	173,510,832	96.39	3747x	85,484,626
Fructose1	Paired-end	174,235,094	171,756,394	98.58	3710x	131,814,606
Fructose2	Paired-end	175,725,256	169,179,500	96.27	3654x	140,600,982
H2CO2.1	Paired-end	106,966,460	102,417,528	95.75	2212x	35,718,104
H1CO2.2	Paired-end	107,825,776	105,217,654	97.58	2272x	38,144,100

[1] Number of paired-end reads after adapter trimming.
[2] Total reads aligned/Total reads no t/rRNA *100.

Ribo-seq data

Library	Type	Total reads sequenced[1]	Total reads sequenced - nonrtRNA[2]	Total reads aligned	Efficiency %[3]	#Reads CDS
CO1	Single-end	74,932,501	13,766,663	12,001,115	89.38	7,627,229
CO2	Single-end	95,785,654	20,547,257	18,289,274	90.96	11,309,310
Fructose1	Single-end	17,354,705	10,432,465	6,108,135	84.33	6,589,503
Fructose2	Single-end	17,836,485	11,324,313	6,997,772	88.64	7,216,032
H2CO2.1	Single-end	46,949,468	4,528,704	2,382,471	80.64	1,501,240
H1CO2.2	Single-end	41,783,500	4,900,815	3,323,343	85.97	1,628,653

[1] Number of single-end reads after adapter trimming.
[2] Number of single-end reads after removing rRNA and tRNA sequences.
[3] Total reads aligned/Total reads no t/rRNA *100.

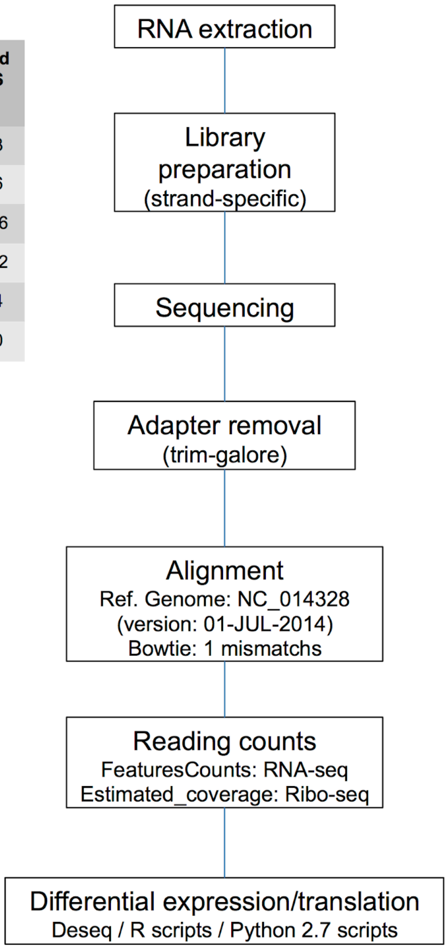

Supplementary Figure 1. RNA-seq and Ribo-seq library sequencing statistics.

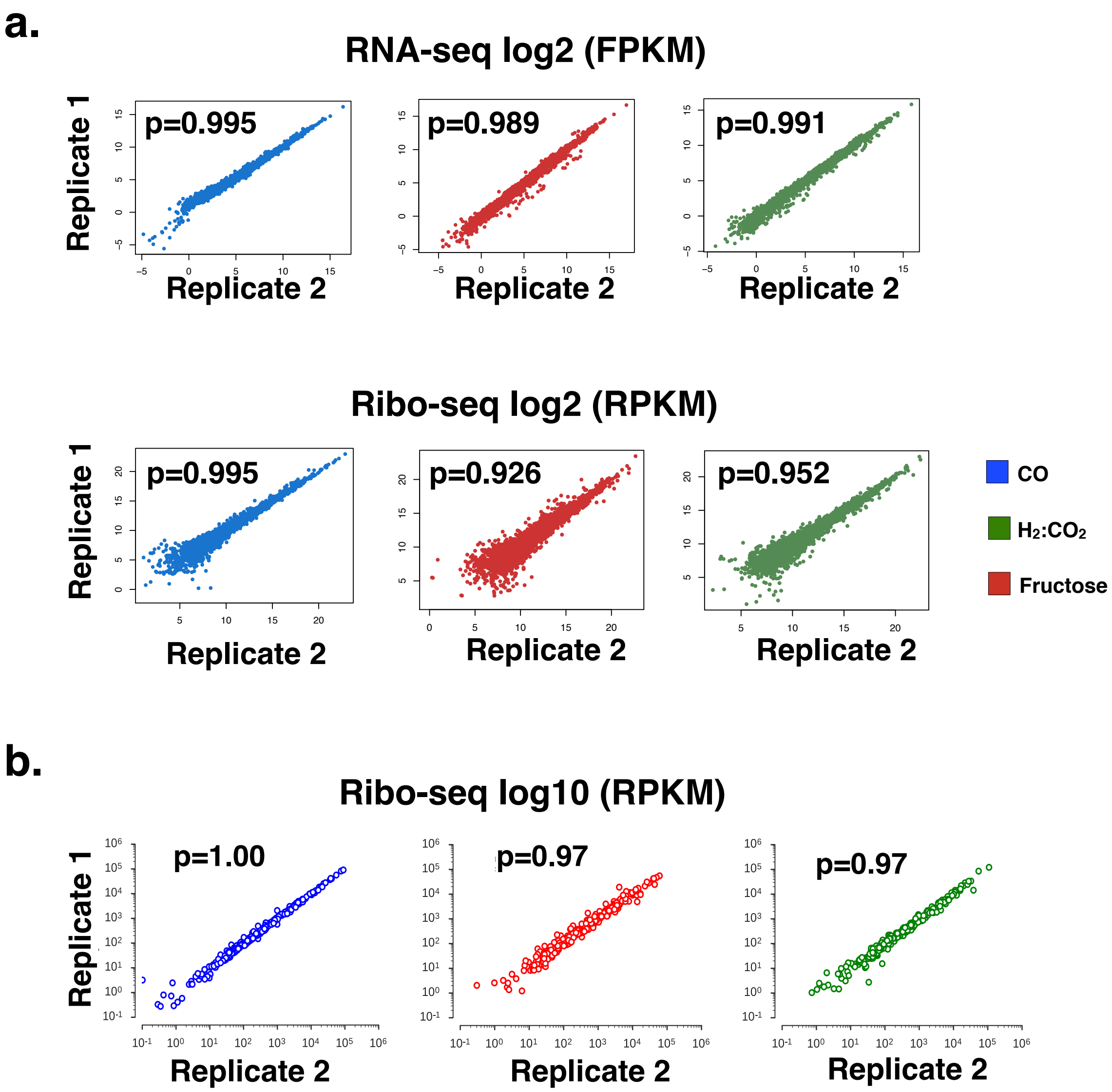

Supplementary Fig. 2 Correlations between RNA-seq and Ribo-seq biological replicates. Pearson's correlations are depicted by "p". (a) Biological replicates correlations at the gene level. **(b)** Biological replicates correlations of Ribo-seq at the RAST subsystem level (Spearman correlations are 1.00, 0.99 and 0.99 for CO, fructose and H_2:CO_2, respectively).

Supplementary Fig. 3

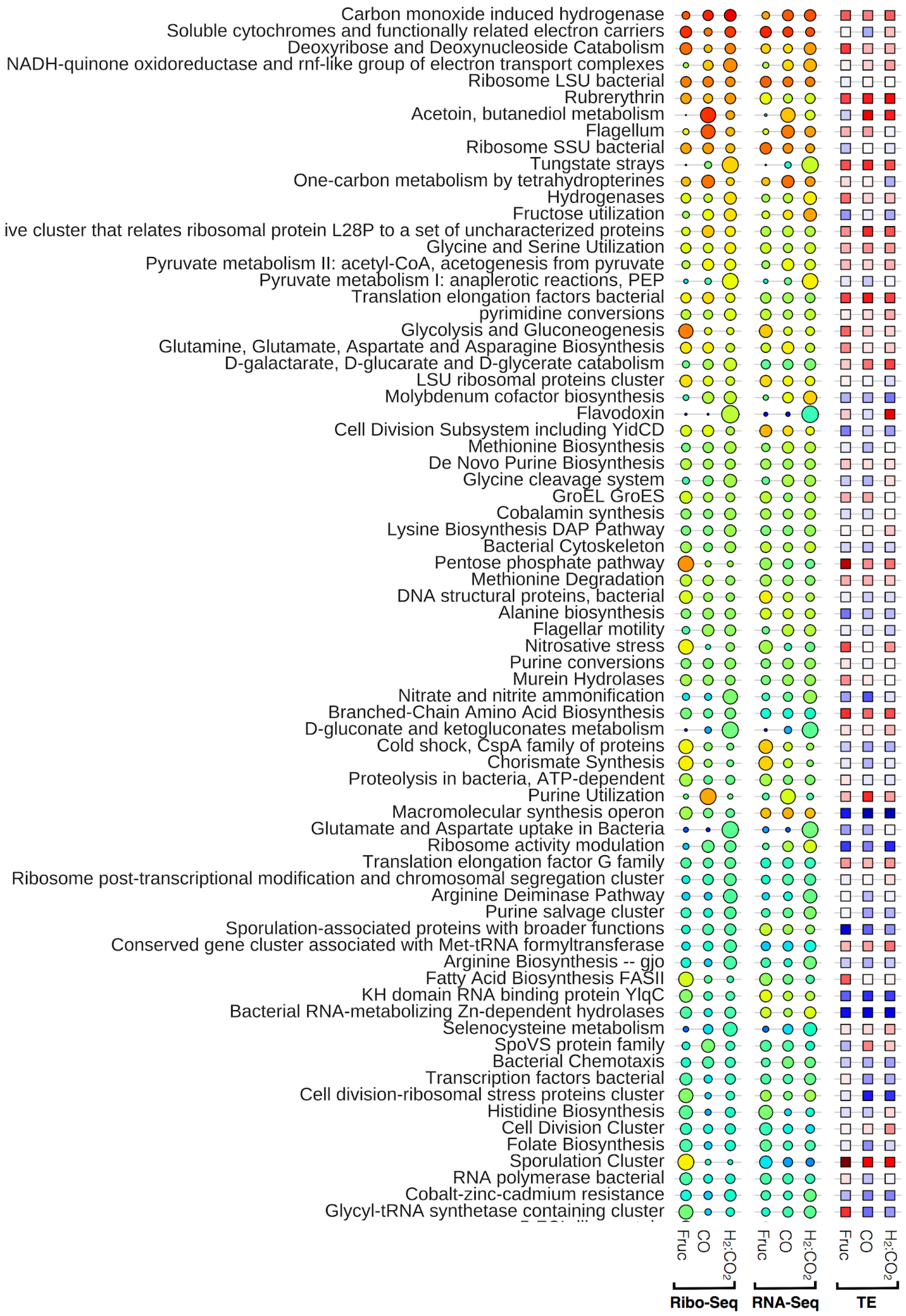

Supplementary Fig. 3 continued

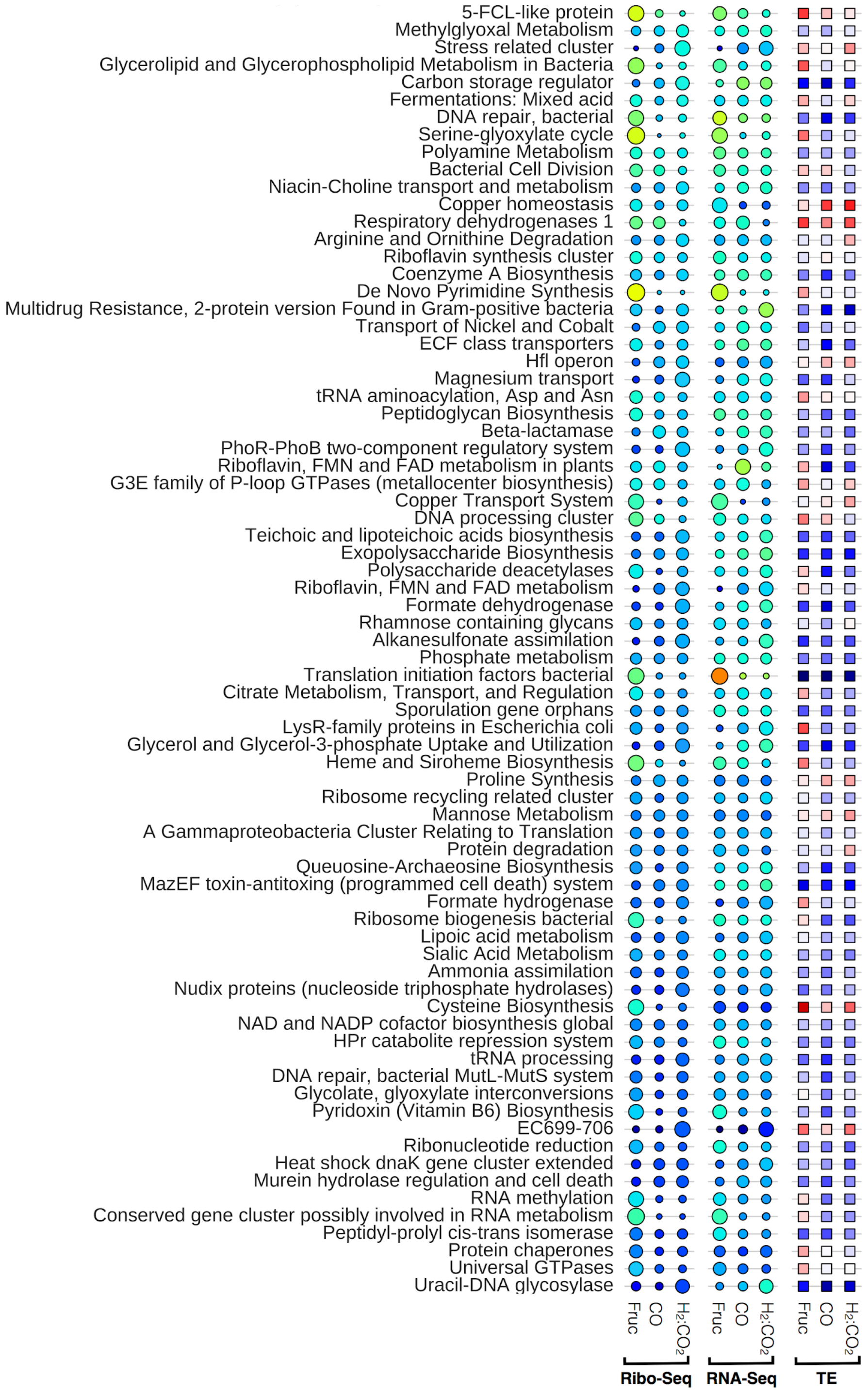

Supplementary Fig. 3 continued

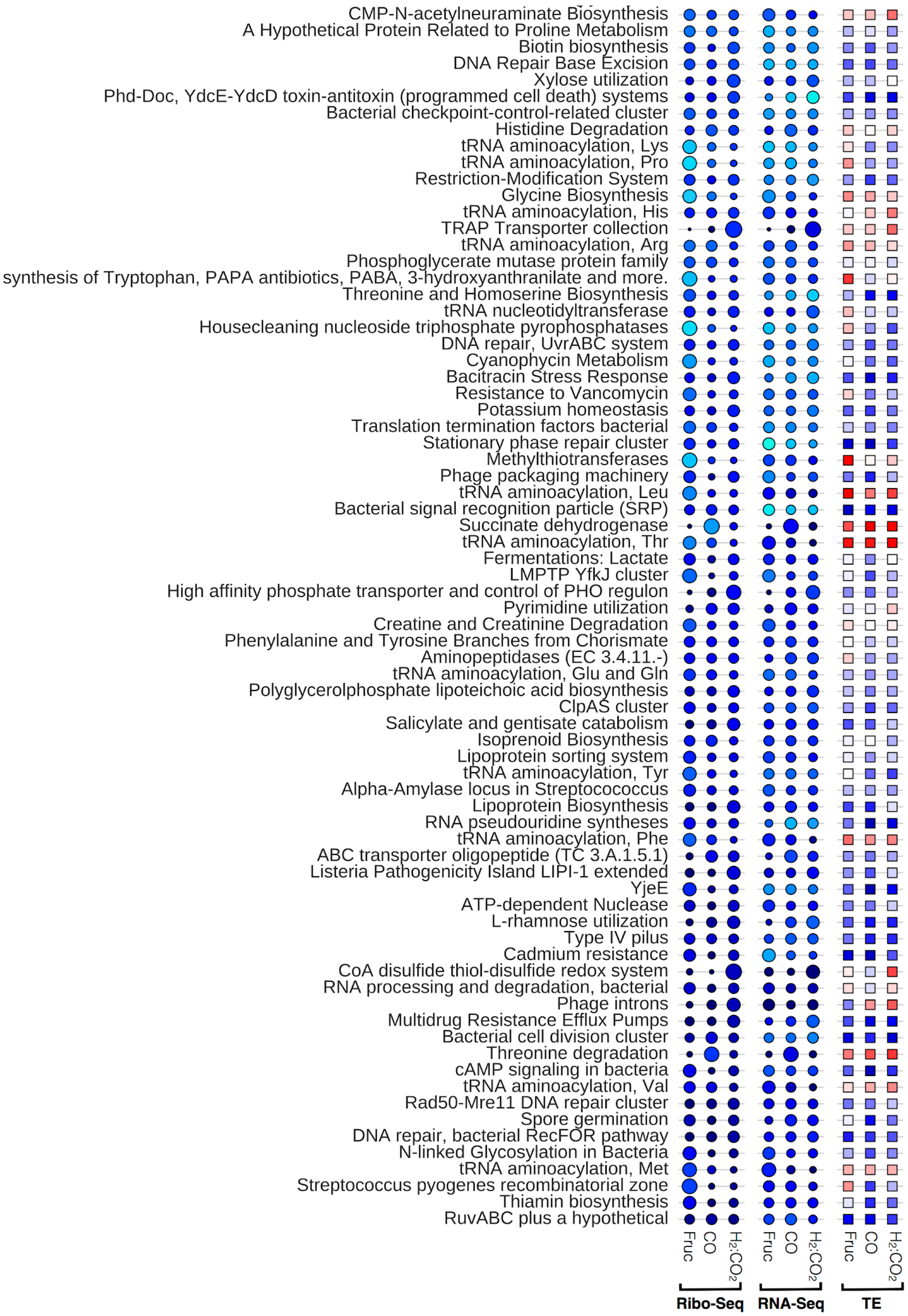

Supplementary Fig. 3 continued

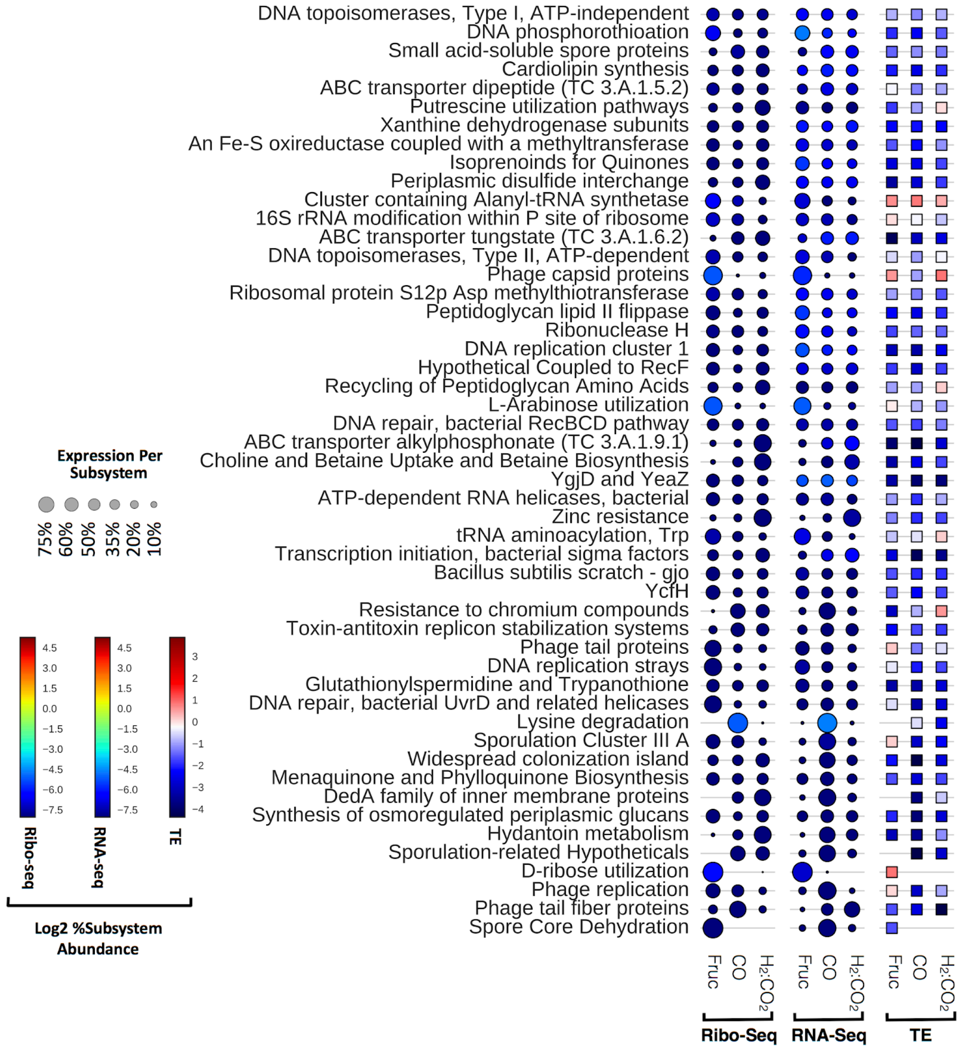

Supplementary Figure 3. Functional enrichment of RAST subsystems in Ribo-seq, RNA-seq and translational efficiency (TE), for fructose (Fruc), CO and H_2:CO_2 growth. Data are sorted descending according to enrichment in H_2:CO_2. Genes were grouped into RAST subsystems and translation and transcription were both percent-normalized per each experiment. Each column represents the level of translation or transcription (% normalized) per each experiment and the bubble color reflects the intensity (% normalized). Each row represents a comparison between the three datasets and the size of bubbles represents the level of translation or transcription per each subsystem (% normalized). TE is compared per each subsystem and depicted by squares.The colors represent percent TE values. All values in the figure are shown as $\log_2$.

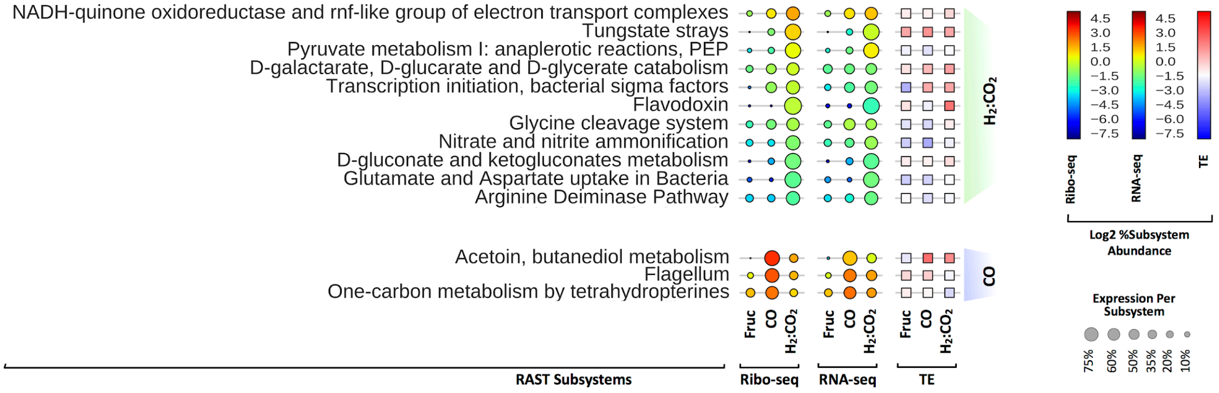

Supplementary Figure 4. Functional enrichment of autotrophic RAST subsystems, using Ribo-seq and RNA-seq data. The subsystems shown are differentially enriched in either H_2:CO_2 (top) or CO (bottom). The Rnf complex, the flavodoxin, and the D-gluconate/ketogluconates subsystems exhibit differentially TE in H_2:CO_2.

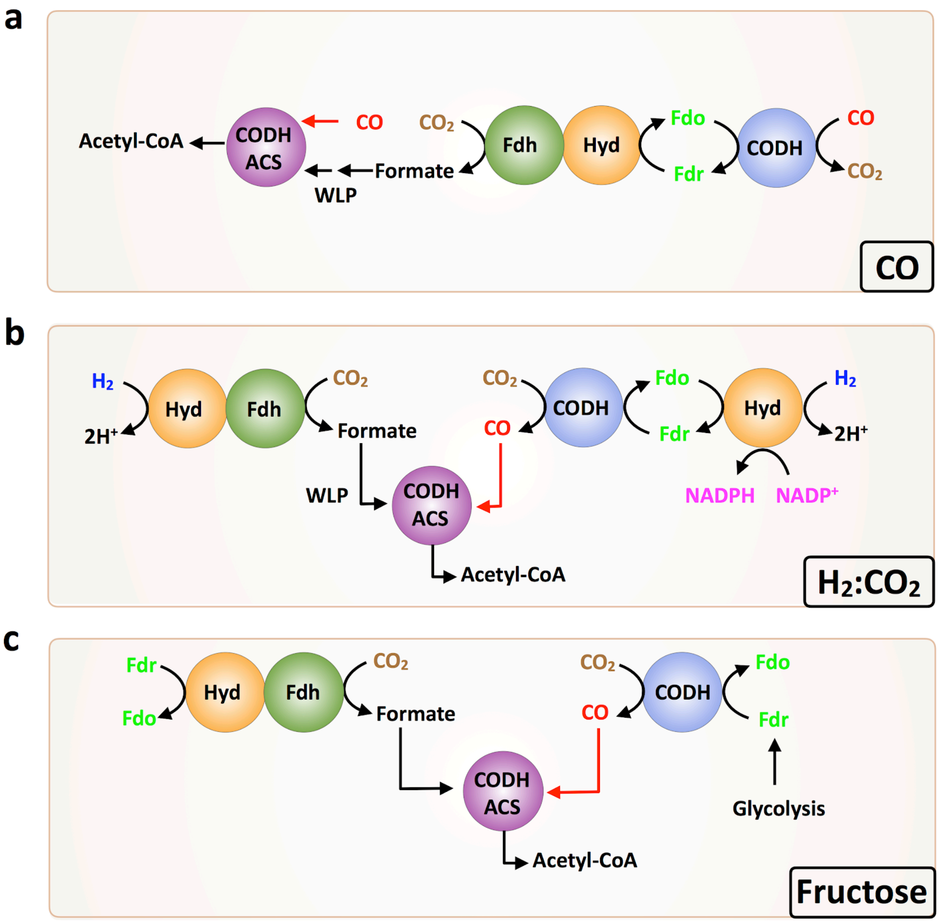

Supplementary Figure 5. Energy conservation involving formate dehydrogenase (Fdh), hydrogenase (Hyd), and carbon monoxide dehydrogenase (CODH/ACS) complexes in (a) CO, (b) H_2:CO_2 and (c) Fructose. Wood-Ljungdahl pathway (**WLP**), Ferredoxin reduced (**Fdr**), Ferredoxin oxidized (**Fdo**).

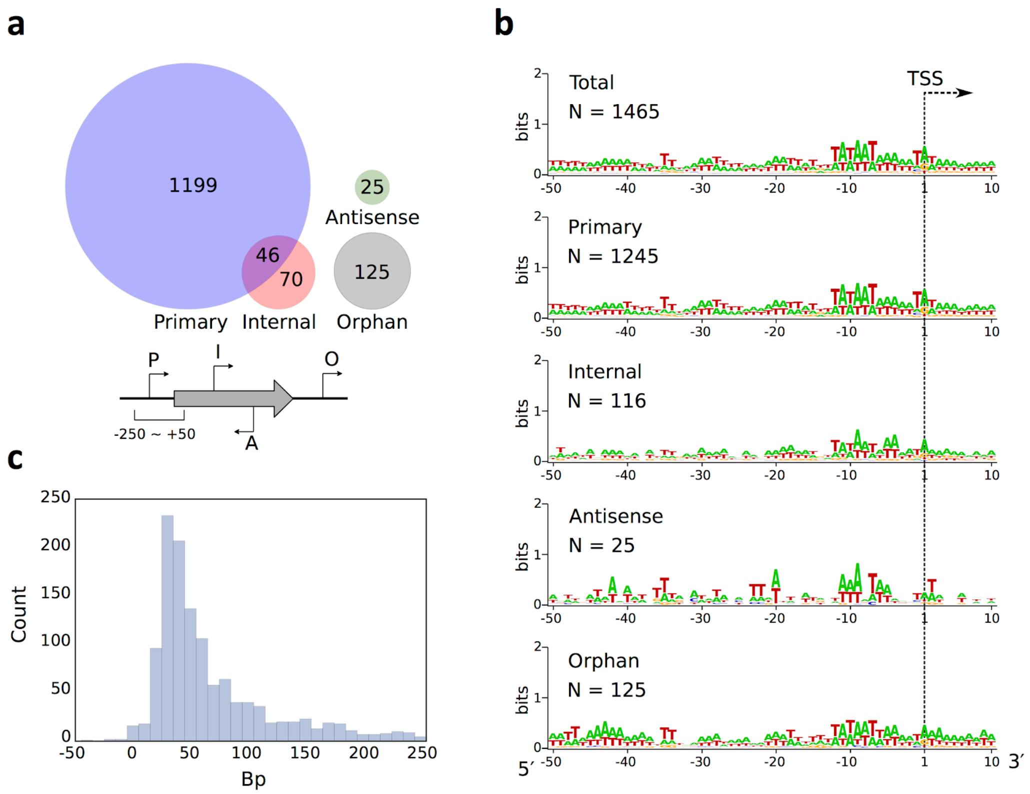

Supplementary Figure 6. Determination of TSS characteristics. (a) Categorization of TSSs by genomic locations. **(b)** Conserved motif sequences of promoters identified by TSS annotation. **(c)** Distribution of 5′UTR lengths.

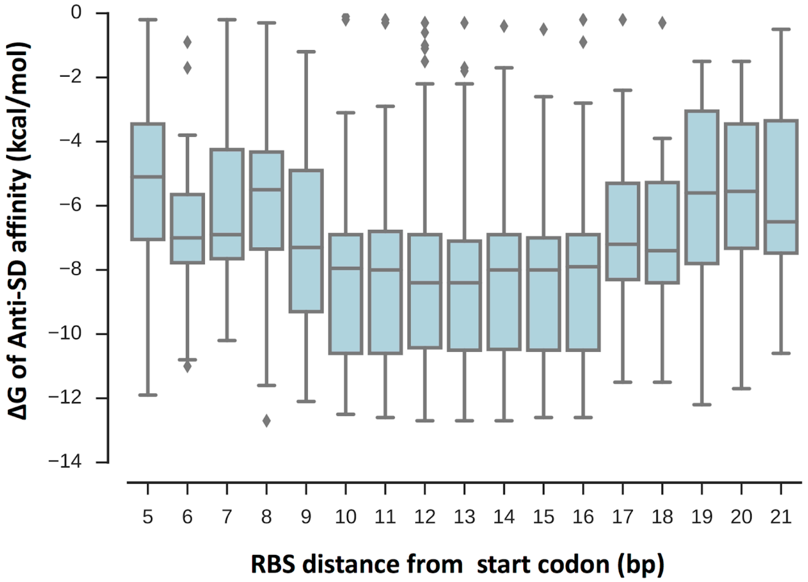

Supplementary Figure 7. Correlation between -ΔG of anti-SD (anti-Shine Dalgarno) and the 5'RBS distance from translation start site.

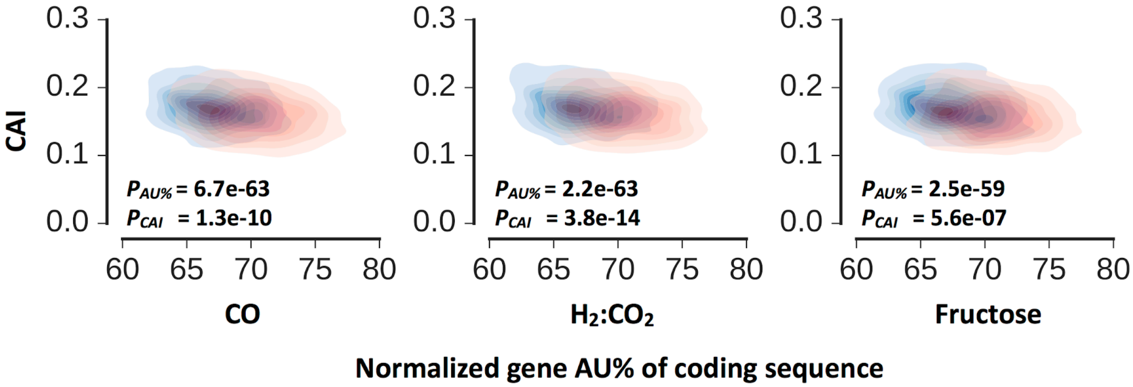

Supplementary Figure 8. Comparison between the codon adaptation index (CAI) and the AU% content in the mRNA coding region in high-TE (blue) and low-TE genes (red) under CO, H_2:CO_2, and fructose growth conditions. The AU% of mRNA has more significant influence on TE over CAI. Mann-Whitney U test was employed to determine P values.

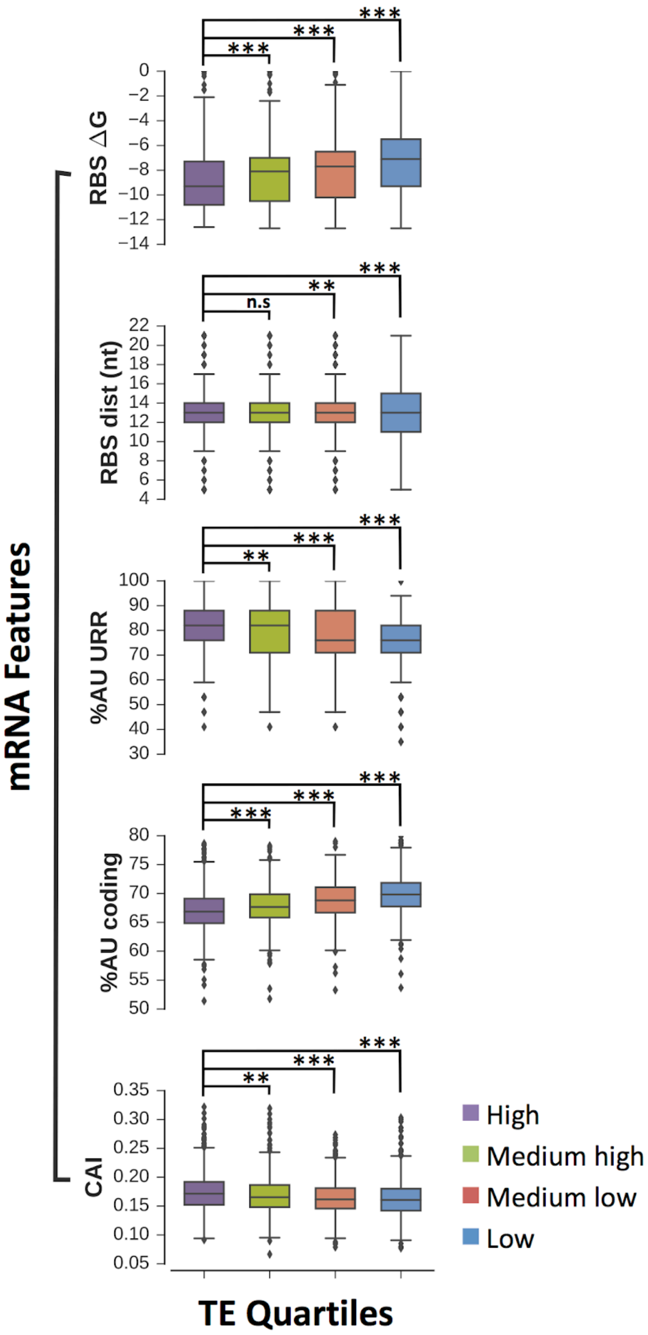

Supplementary Figure 9. Comparisons of the 5′UTR and the coding region features in genes grouped by their TE_{max} values in four color-coded quartiles. Each quartile has 870 genes. The ranges of TE_{max} cutoffs are 0.01-0.32 (Low, blue color), 0.32-0.63 (Medium Low, red color), 0.63-1.18 (Medium High, green color) and 1.18-4.97 (High, violet color). Levene's test was used to calculate the *P* values for the variability in the RBS distances from the translation start codon. Mann-Whitney U test was used for other comparisons. *P* values $<1e^{-4}$ are shown above each of boxplot sets as "***". *P* values <0.01 and $\geq 1e^{-4}$ are indicated with "**" and n.s. indicates not significant difference.

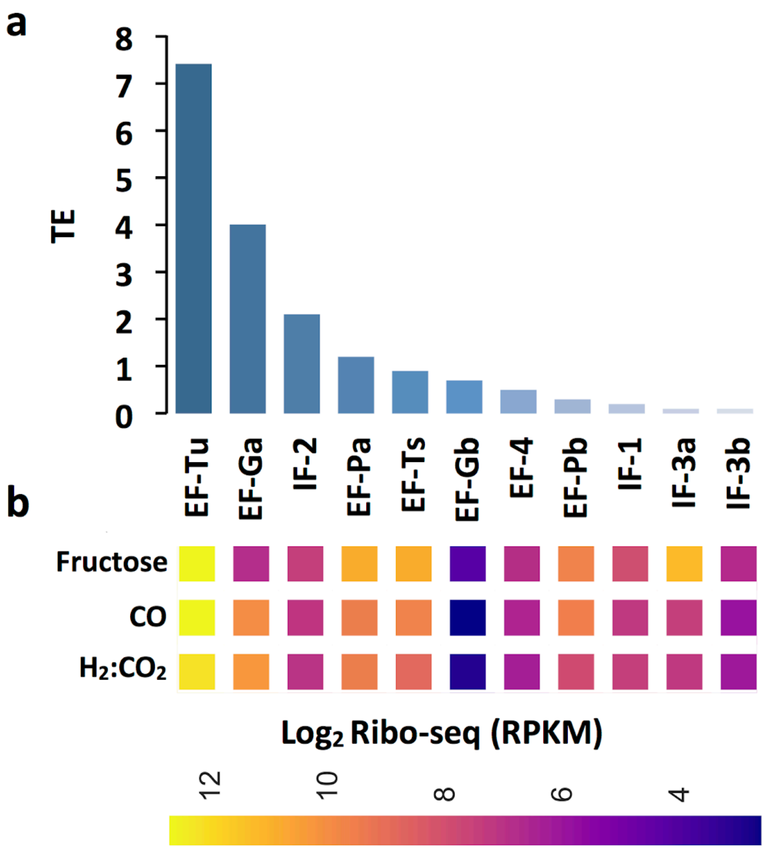

Supplementary Figure 10. Maximum translational efficiency (TEmax) and the translation of each of the factors involved in translation initiation and translation elongation. (a) TE_{max} of each gene. Two 99.75% identical genes (*Clju_c41200* and *Clju_c41060*) encode EF-Tu. Two 27% identical loci (*Clju_c24640* encodes EF-Ga and *Clju_c41380* encodes EF-Gb) encode EF-G. Two 24% identical loci (*Clju_c41070* encodes EF-Pa and is larger than EF-Pb, which is encoded by *Clju_c11010*) encode EF-P. **(b)** The log_2 translation level of each of the translation factors in all growth conditions.

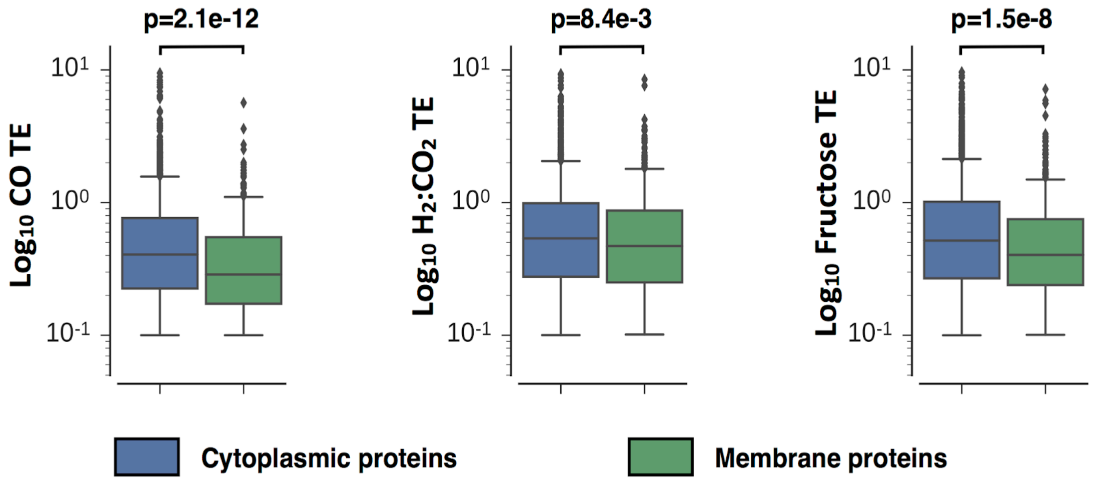

Supplementary Figure 11. Comparison between the translational efficiency of membrane and cytoplasmic proteins. Membrane proteins were predicted using the TMHMM Server v. 2.0. The *P* values were calculated using the Mann-Whitney U test. Total number of predicted membrane proteins were 782 versus 3374 cytoplasmic proteins.

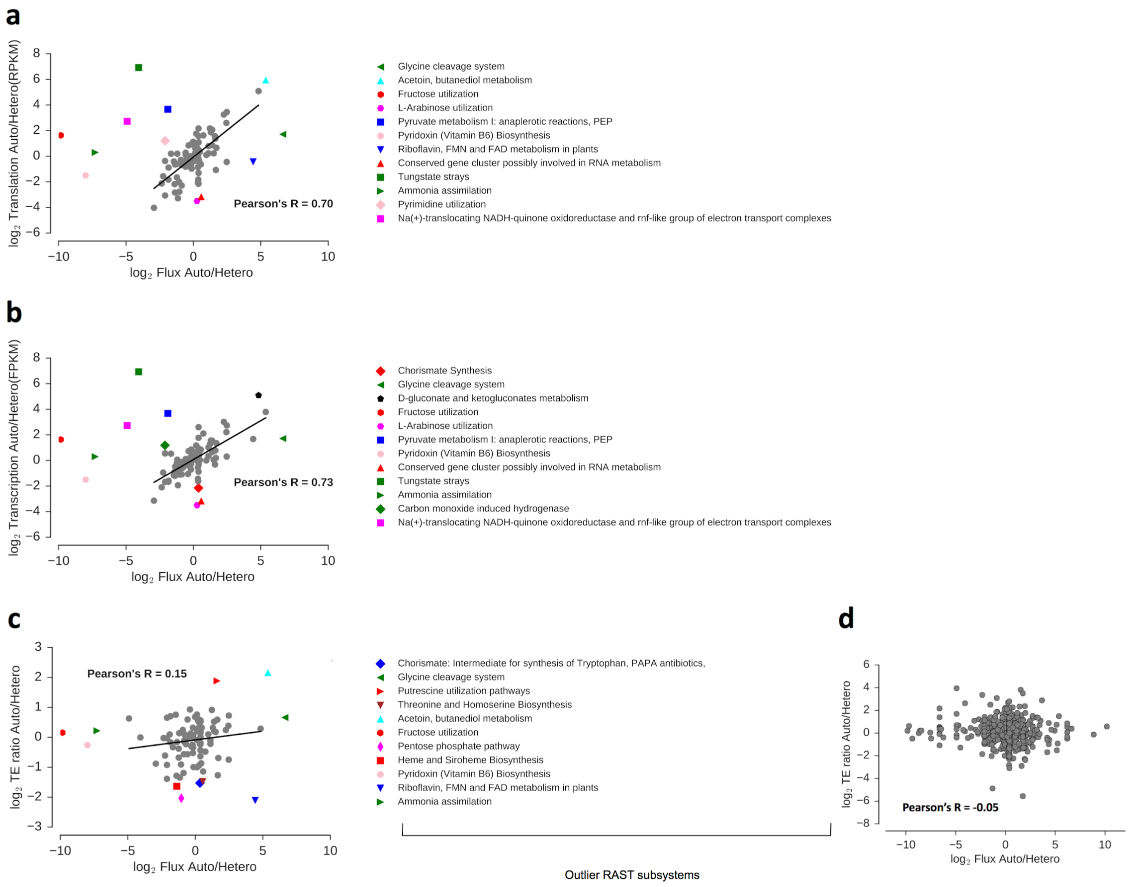

Supplementary Figure 12. Predicted flux correlation with Ribo-seq, RNA-seq and translation efficiency. (a) Correlation is between the log_2 ratio of H_2:CO_2/Fructose predicted fluxes and log_2 H_2:CO_2/Fructose Ribo-seq (RPKM) values. The grey dots represent RAST subsystems that were used to fit the linear regression line and also used to calculate Pearson's correlation. Outlier subsystems are colored and are annotated on the right panel. **(b)** Correlation is between the log_2 ratio of H_2:CO_2/Fructose predicted fluxes and log_2 H_2:CO_2/Fructose RNA-seq (FPKM) values. The grey dots represent RAST subsystems that were used to fit the linear regression line and also used to calculate Pearson's correlation. Outlier subsystems are colored and are annotated on the right panel. **(c)** Correlation is between the log_2 ratio of H_2:CO_2/Fructose predicted fluxes and log_2 H_2:CO_2/Fructose TE values. The grey dots represent RAST subsystems that were used to fit the linear regression line and also used to calculate Pearson's correlation. Outlier subsystems are colored and are annotated on the right panel. **(d)** Correlation is between log_2 ratio of H_2:CO_2/Fructose predicted fluxes and log_2 H_2:CO_2/Fructose TE values at the gene level. Pearson's correlation was calculated without outlier removal.

Supplementary Table 1. Proteomic analysis of the Rnf complex values represent normalized relative abundance

Protein	Fructose R1	Fructose R2	H_2:CO_2 R1	H_2:CO_2 R2	Fructose Ave	H_2:CO_2 Ave
**RseC**	665.3	371.0	2953.8	1481.2	518.2	2217.5
**RnfC**	397.8	536.9	1755.7	2488.3	467.4	2122.0
**RnfD**	274.2	600.5	947.0	3128.4	437.4	2037.7
**RnfG**	513.6	686.5	2099.7	2004.2	600.1	2051.9
**RnfE**	958.4	1194.2	1290.8	1573.0	1076.3	1431.9
**RnfB**	441.3	517.3	2653.5	1864.0	479.3	2258.7

Supplementary Table 2. Effect of RBS features on TE in Figure 5. Mann-Whitney U test p-values <0.05 are considered significant.

Growth Condition	RBS ΔG†	Distance from start codon*	URR AT%†
**CO**	1.80E-10	1.40E-07	3.70E-06
**H_2:CO_2**	2.90E-09	2.40E-04	2.80E-06
**Fructose**	2.30E-07	0.011	1.20E-04

† Comparisons are between 20th (low) vs 80th (high) percentiles
* Comparisons are between 15 & 85th (low) vs 50th (high) percentiles (see Figure 5f)

Supplementary Table 3. Gene counts per TE_{max} quartile per RAST category and Fisher tests.

RAST Category	Gene counts per TE_{max} Quartile					P value
	Low	Medium low	Medium high	High	TE rank†	
**Carbohydrates**	19	21	46	53	H	0.009
**Nitrogen Metabolism**	3	3	9	18	H	0.002
**Nucleosides**	12	25	23	38	H	0.020
**Respiration**	12	6	11	26	H	0.003
**Amino Acids**	23	37	66	60	MH	0.009
**Cell Wall**	22	32	25	17	ML	0.015
**DNA Metabolism**	24	29	12	9	ML/L	0.002 / 0.006
**RNA Metabolism**	25	28	22	16	L	0.043
**Dormancy**	12	7	5	5	L	0.006
**Regulation**	8	6	4	2	L	0.029
**Virulence**	26	19	15	19	L	0.003
**Cell Division and Cell Cycle**	5	6	9	4	NS	>0.05
**Cofactors, Vitamins,**	33	39	57	62	NS	>0.05

**Prosthetic Groups, Pigments**						
**Fatty Acids, Lipids, and Isoprenoids**	13	9	10	14	NS	>0.05
**Membrane Transport**	20	24	28	17	NS	>0.05
**Miscellaneous**	5	0	8	7	NS	>0.05
**Motility and Chemotaxis**	14	20	11	12	NS	>0.05
**Protein Metabolism**	18	39	53	55	NS	>0.05
**Stress Response**	6	5	5	6	NS	>0.05
**Metabolism of Aromatic Compounds**	0	2	1	0	U	na
**Phages, Prophages, Transposable elements, Plasmids**	1	2	5	4	U	na
**Phosphorus Metabolism**	5	5	2	2	U	na
**Potassium metabolism**	0	2	2	0	U	na
**Secondary Metabolism**	0	0	0	3	U	na
**Sulfur Metabolism**	2	0	3	2	U	na

† Fisher Test significance, **H**: Significant in the High quartile, **MH**: Significant in Medium High quartile, **ML**: Significant in Medium Low quartile, **L**: Significant in Low quartile, **NS**: Not significant in any quartile**, U**: Under the sufficient number to run test.

Supplementary Methods

1. Flux balance analysis

Our previously reconstructed model for *C. ljungdahlii* (*i*NH637)[1] was used as platform to understand the metabolism of *C. ljungdahlii* at various growth conditions[2]. Experimental measurements of RNA-seq and Ribo-seq under autotrophic and heterotrophic conditions were collected under exponential growth phase. Both data sets were compared with predicted flux distributions under autotrophic and heterotrophic conditions. Genome-scale model simulations were performed using the Gurobi Optimizer Version 5.6.3 (Gurobi Optimization Inc., Houston, Texas) solver in MATLAB (The MathWorks Inc., Natick, MA) with the COBRA Toolbox[3]. Flux balance analysis (FBA) was used to simulate the genome-scale model[4].

The amplitude of metabolic fluxes was determined using random sampling. To uniformly sample the solution space of *i*NH637, the model was reduced[5] and optGpSampler[6] for MATLAB with Gurobi Optimizer Version 6.5.0 was performed.

Genes with zero flux were removed and remaining genes were grouped into RAST subsystems. The log2 ratio of H_2:CO_2/Fructose RAST subsystem flux was plotted against the log2 ratio of H_2:CO_2/Fructose RAST subsystem TE, RNA-seq and Ribo-seq. Outliers were removed in two steps. The first step involved removal of subsystems that had over three standard deviations above or below the mean. Step two involved fitting a linear regression line and removal of 2% of biggest outliers in three iterations.
The amplitude of metabolic fluxes was determined using FVA[7]. Reactions carrying an absolute flux lower than 10 were set to zero. All reactions that could not carry flux under autotrophic and heterotrophic conditions were identified and removed from the models[5]. To uniformly sample the solution space of iNH637, optGpSampler for MATLAB with Gurobi Optimizer Version 6.5.0 was used[6]. The reduced models were sampled with 50,000 sample points.

2. Shotgun proteomics

Protein Preparation

Cells were pelleted and lysed in a homemade lysis buffer containing 3% SDS, 75 mM sodium chloride, 1 mM sodium fluoride, 1 mM beta-glycerophosphate, 1 mM sodium orthovanadate, 10 mM sodium pyrophosphate, 1 mM phenylmethylsulfonyl fluoride and 1 cOmplete™, Mini EDTA-free Protease Inhibitor Cocktail (Roche). To ensure complete lysis, lysates were sonicated using a probe sonicator for 10 seconds at 35% intensity followed by a 10 second rest and repeated three times. Cellular debris was pelleted by centrifuging lysates at 16,000 x g for 10 minutes at 4°C and the resulting supernatant was retained for downstream analysis. An equal volume of 8 M Urea was added to each sample to facilitate protein denaturation.Protein disulfide bonds were reduced with dithiothreitol and alkylated with iodoacetamide as previously described[8]. Reduced and alkylated proteins were precipitated by adding trichloroacetic acid at a ratio of 1:4 with sample volume and incubating the samples on ice for 10 min. Precipitated proteins were pelleted by centrifugation at 16,000 x g for 10 minutes at 4°C, and washed two times with ice-cold acetone using the same spin conditions. Precipitated proteins were re-suspended in 1 M urea and 50 mM HEPES, pH 8.5 for proteolytic digestion. Digestion was performed in a two-step process as previously described[9]. Following digestion, peptides were desalted using C18 solid-phase extraction columns as previously described[10]. Desalted peptides were dried in a speed vacuum and quantified using the Pierce™ Quantitative

Colorimetric Peptide Assay (Thermo). 50 ug of each sample was aliquoted and dried for proteomic analysis.

Peptides were labeled with tandem mass tag (TMT) reagents (Thermo) as previously described[9]. Following labeling, the samples were mixed and fractionated using Pierce™ High pH Reversed-Phase Peptide Fractionation Kit following manufacturer instructions. Fractions were again dried and re-suspended in 5% formic acid/5% acetonitrile prior to LC-MS2/MS3 analysis.

LC-MS2/MS3 Analysis

LC-MS2/MS3 analysis was performed using an Oribtrap Fusion mass spectrometer (Thermo) with an in-line Easy-nLC 1000 (Thermo). Peptide samples were introduced into the instrument through a home-pulled, home-packed column (inner diameter: 100 µm, outer diameter: 360 µm; media: 0.5 cm of C4 resin [5 µm], 0.5 cm of C18 resin [3 µm] and 30 cm of C18 resin [1.8 µm]). Peptides were eluted from the column with a linear gradient of 11% to 30% acetonitrile in 0.125% formic acid over 165 minutes at a flow rate of 300 nL/minute. The column was heated to 60 °C and electrospray ionization was achieved by applying 2000 V through a stainless steel "T" junction at the inlet of the column.

The mass spectrometer was operated in data-dependent mode with parameters previously described[9]. Briefly, a survey scan over the mass to charge (m/z) range of 500-1200 was performed in the Oribitrap at a resolution of 120000. The most abundant ions were selected for MS2 and MS3 analysis. For MS2, ions were isolated in the quadrupole, fragmented with collision-induced dissociation and analyzed in the ion trap. For MS3 analysis, synchronous precursor selection was used to select a maximum of 10 MS2 ions which were then fragmented using higher energy collisional dissociation. Fragment ions were analyzed in the Orbitrap at a resolution of 60000. All data collected was centroided.

Data Analysis

Raw mass spectra were processed using Proteome Discoverer 2.1.0.81 (Thermo). The SequestHT[11] node was used to assign MS2 spectra to the *Clostridium ljungdahlii* ATCC 55383 database (Uniprot: downloaded 02/26/18). The database was appended to include a decoy database of all the protein sequences in reverse order for subsequent false discovery rate (FDR) estimation[12,13]. Search tolerances were set to 50 ppm for MS1 scans and 0.6 Da for MS2 scans. Static modifications of carbamidomethylation of cysteines, TMT10plex tags on lysine and peptide n-termini and variable oxidation of methionine were specified. Enzyme specificity was set to full trypsin digest with a maximum of two missed

cleavages allowed per peptide. Data were filtered to a <1% FDR at the peptide and protein level[12]. TMT reporter ion intensities were extracted from the MS3 spectra for quantitative analysis. Data were filtered, summed and normalized as previously described[9].

Supplementary References

1. Nagarajan, H. *et al.* Characterizing acetogenic metabolism using a genome-scale metabolic reconstruction of Clostridium ljungdahlii. *Microb. Cell Fact.* **12,** 118 (2013).
2. Tan, J., Zuniga, C. & Zengler, K. Unraveling interactions in microbial communities - from co-cultures to microbiomes. *Journal of Microbiology* **53,** 295–305 (2015).
3. Schellenberger, J. *et al.* Quantitative prediction of cellular metabolism with constraint-based models: The COBRA Toolbox v2.0. *Nat. Protoc.* **6,** 1290–1307 (2011).
4. Orth, J. D., Thiele, I. & Palsson, B. Ø. What is flux balance analysis? *Nat. Biotechnol.* **28,** 245–248 (2010).
5. Zuniga, C. *et al.* Predicting dynamic metabolic demands in the photosynthetic eukaryote Chlorella vulgaris. *Plant Physiol.* pp.00605.2017 (2017). doi:10.1104/pp.17.00605
6. Megchelenbrink, W., Huynen, M. & Marchiori, E. optGpSampler: An improved tool for uniformly sampling the solution-space of genome-scale metabolic networks. *PLoS One* **9,** (2014).
7. Mahadevan, R. & Schilling, C. H. The effects of alternate optimal solutions in constraint-based genome-scale metabolic models. *Metab. Eng.* **5,** 264–276 (2003).
8. Haas, W. *et al.* Optimization and use of peptide mass measurement accuracy in shotgun proteomics. *Mol. Cell. Proteomics* **5,** 1326–1337 (2006).
9. Lapek, J. D., Lewinski, M. K., Wozniak, J. M., Guatelli, J. & Gonzalez, D. J. Quantitative Temporal Viromics of an Inducible HIV-1 Model Yields Insight to Global Host Targets and Phospho-Dynamics Associated with Protein Vpr. *Mol. Cell. Proteomics* **16,** 1447–1461 (2017).
10. Tolonen, A. C. & Haas, W. Quantitative proteomics using reductive dimethylation for stable isotope labeling. *J. Vis. Exp.* (2014). doi:10.3791/51416
11. Eng, J. K., Mccormack, A. L. & Yates, J. R. An Approach to Correlate Tandem Mass Spectral Data of Peptides with Amino Acid Sequences in a Protein

Database. *Am. Soc. Mass Spectrom.* **5,** 976–989 (1994).

12. Elias, J. E., Haas, W., Faherty, B. K. & Gygi, S. P. Comparative evaluation of mass spectrometry platforms used in large-scale proteomics investigations. *Nat. Methods* **2,** 667–675 (2005).

13. Elias, J. E. & Gygi, S. P. Target-decoy search strategy for increased confidence in large-scale protein identifications by mass spectrometry. *Nat. Methods* **4,** 207–214 (2007).
