# Supplementary material for: Optimization of carbon and energy utilization through differential translational efficiency
Source: Nat Commun. 2018 Oct 26;9:4474. doi: 10.1038/s41467-018-06993-6 (PMC6203783; doi:10.1038/s41467-018-06993-6)
Supplement: Supplementary file 3 — Description of Additional Supplementary Files [file 41467_2018_6993_MOESM3_ESM.pdf]

Description of Additional Supplementary Files

File Name: Supplementary Data 1
Description: RAST annotation and omics data at the subsystem level.

File Name: Supplementary Data 2
Description: Merged database specifying genetic features and their coordinates on the genome, Ribo-seq (RPKM), RNA-seq (FPKM) and TE values, and DESeq2 expression fold change and adjusted P values (Padj).

File Name: Supplementary Data 3
Description: List of all transcription start sitess (TSSs) of C. ljungdahlii identified in this study. Nucleotide position in column B is taken from RefSeq Accession No. NC_014328.1. Column C-E indicate the genes corresponding to primary TSSs, antisense TSSs and internal TSSs, respectively. Orphan TSSs are marked as '+' in column F. Column G indicates UTR length calculted by the nucleotide distance between primary TSSs and start condon of corresponding genes. Column I indicates confidence score of TSS annotation (See Methods).

File Name: Supplementary Data 4
Description: Flux balance predictions for H2:CO2 and Fructose growth with RAST subsystem annotations.
